# A comparison of calibration data from full field digital mammography units for breast density measurements

**DOI:** 10.1186/1475-925X-12-114

**Published:** 2013-11-09

**Authors:** Erin EE Fowler, Beibei Lu, John J Heine

**Affiliations:** 1 Division of Population Science, H. Lee Moffitt Cancer Center & Research Institute, 12902 Magnolia Drive, Tampa, FL 33612, USA

**Keywords:** Breast density, Calibration, Phantom imaging, Direct x-ray conversion, Full field digital mammography

## Abstract

**Background:**

Breast density is a significant breast cancer risk factor measured from mammograms. The most appropriate method for measuring breast density for risk applications is still under investigation. Calibration standardizes mammograms to account for acquisition technique differences prior to making breast density measurements. We evaluated whether a calibration methodology developed for an indirect x-ray conversion full field digital mammography (FFDM) technology applies to direct x-ray conversion FFDM systems.

**Methods:**

Breast tissue equivalent (BTE) phantom images were used to establish calibration datasets for three similar direct x-ray conversion FFDM systems. The calibration dataset for each unit is a function of the target/filter combination, x-ray tube voltage, current × time (mAs), phantom height, and two detector fields of view (FOVs). Methods were investigated to reduce the amount of calibration data by restricting the height, mAs, and FOV sampling. Calibration accuracy was evaluated with mixture phantoms. We also compared both intra- and inter-system calibration characteristics and accuracy.

**Results:**

Calibration methods developed previously apply to direct x-ray conversion systems with modification. Calibration accuracy was largely within the acceptable range of ± 4 standardized units from the ideal value over the entire acquisition parameter space for the direct conversion units. Acceptable calibration accuracy was maintained with a cubic-spline height interpolation, representing a modification to previous work. Calibration data is unit specific, can be acquired with the large FOV, and requires a minimum of one reference mAs sample. The mAs sampling, calibration accuracy, and the necessity for machine specific calibration data are common characteristics and in agreement with our previous work.

**Conclusion:**

The generality of our calibration approach was established under ideal conditions. Evaluation with patient data using breast cancer status as the endpoint is required to demonstrate that the approach produces a breast density measure associated with breast cancer.

## Introduction

Mammographic breast density is a significant breast cancer risk factor [1-3]. Although used extensively in research, breast density is not generally used in the clinical environment for breast cancer risk applications [4] due in large part to the lack of an automated measurement. There are various methods under evaluation for estimating breast density from either raw or calibrated mammograms [5]. A large portion of breast density research was derived without calibration [1,2], as calibration is a more recent development for mammography.

Ideally, calibration adjusts for inter-patient x-ray image acquisition technique differences to produce some form of standardized data representation [6-9]. Calibration research is still in its early stage of development and there are few published reports evaluating its potential application relative to the volume of published breast density research using raw mammograms. The findings from calibration research have been mixed in identifying a measure that strengthens the associations with breast cancer in comparison with the operator-assisted percentage of breast density measure [10-15]. Due to its stage of development, it may be premature to conclude whether calibration is generally a useful technique for risk assessments. However, one benefit of establishing a calibration method is that it permits automated breast density measurements. We have posited that calibration may be an important step for automation.

Full field digital mammography (FFDM) detector technologies can be broadly categorized as either indirect or direct x-ray conversion systems [16]. Although these designs have many characteristics that vary, until recently both technologies produced an energy weighted integrated signal at the pixel level [17]. More recently, another type of direct x-ray conversion technology was approved for clinical use in the US that uses photon counting detection technology [18], which, in contrast to the established FFDM designs, does not produce an integrated weighted signal. Currently, it is not known if calibration will produce equivalent findings across these varying FFDM platforms.

We applied a calibration methodology developed previously for a General Electric Senographe 2000D FFDM system [19-22], which is an indirect x-ray conversion technology. Our findings based on images taken from this technology [12-14] suggest that calibrated breast density measurements are strong indicators of risk, providing justification to investigate the merits of calibration in more detail. As many characteristics vary between the direct and indirect x-ray conversion systems, the applicability of our calibration methodology has yet to be established for direct x-ray conversion FFDM systems.

In this current report, we expand our understanding of calibration gained previously [21,22] and establish a calibration system for a direct x-ray conversion FFDM design using phantom images acquired from three Hologic Selenia FFDM units, as the primary analysis. We considered several design objectives. One objective is to minimize the amount of calibration data collection while maintaining acceptable calibration accuracy, representing an important compromise. Although optimal, it is nearly impossible to sample all acquisition technique combinations to construct the calibration curves. Therefore, some form of sampling scheme and interpolation methodology must be established to minimize effort while maintaining acceptance accuracy. It is reasonable to assume that if calibration requires excessive phantom imaging effort or is difficult to apply across imaging platforms without considerable modification, it may not be used beyond research. Another objective is to evaluate whether calibration data collected from one FFDM unit can be applied to another similarly manufactured unit, with or without modification, as inter-unit generalization for a given technology is an important step for universal application. As a secondary objective, we also compared calibration and detector response data obtained from the Hologic units investigated in this report with those previously acquired from the General Electric FFDM unit when applicable to assess inter-technology similarities.

## Methods

We acquired calibration and exposure response data from three Hologic Selenia FFDM units to evaluate the generality of our approach. Calibration curves were generated by imaging standard breast tissue equivalent (BTE) phantoms (CIRS, Norfolk, VA) described previously [22]. Our BTE phantom set includes 100% fibroglandular (glandular) and 100% adipose BTE materials that are of 1 mm, 2 mm, 1 cm, and 2 cm thicknesses (i.e. precise heights) and 18 cm × 24 cm in area dimension. These phantoms were combined (stacked) to produce desired composite proportions at a given total thickness (height). For example, combining a 2 cm thickness glandular phantom with a 2 cm thickness adipose phantom gives a 50% glandular composition with a total height of 4 cm. Calibration curves are functions of the compressed breast thickness above the breast support surface, referenced as height, and several other acquisition technique parameters, including target/filter combination, x-ray tube voltage (kV), current × time (mAs), and detector field of view (FOV), representing a five dimensional parameter space. As previously, we refer to the initial data collection as the baseline (BL) calibration dataset. A BL dataset was established for each unit.

The three Selenia systems evaluated in this report are located within the breast clinics at the Moffitt Cancer Center and are used for both screening and diagnostic purposes. Two of these systems, referred to as the H_1_ and H_2_, have a tungsten (W) target with rhodium (Rh) and silver (Ag) filter options. The third unit has a molybdenum (Mo) target with Mo and Rh filter options and is referred to as H_3_. The Selenia detector has 70 micron pitch (pixel spatial resolution), and the raw data used for this work has 14 bit per pixel dynamic range. Two detector FOVs are used for screening mammograms on these units depending upon the choice of compression paddle: 24 cm × 29 cm (large) and 18 cm × 24 cm (small). The General Electric Senographe 2000D FFDM unit is referred to as GE in the report. This unit has a Mo target with Mo and Rh filter options, and a Rh target with a Rh filter. The GE detector has 100 micron spatial resolution, a 19.2 cm × 23 cm detector FOV (i.e. 1914 × 2294 pixels) and 14 bit dynamic range per pixel for the raw data used in this work. As a standard convention, we acquired all phantom images as left cranial caudal (LCC) views. In the LCC view, the detector left border in the vertical direction is parallel with the chest wall position as observed in a displayed image.

The aims of this study were to assess the pixel value – detector exposure (detector response) relationship without attenuation, generate and assess the calibration curves for linearity, and evaluate the calibration accuracy. To minimize the BL data collection, we evaluated the calibration accuracy under these conditions: (a) when applying interpolation for the height variable; (b) when applying a data reduction step to reduce mAs sampling; and (c) as a function of FOV. To evaluate the FOV impact, we acquired the calibration datasets with the large detector FOV only. The validity of collecting calibration data with the large FOV only was evaluated by examining calibration accuracy for images acquired with the small FOV. We made direct comparisons between H_1_ and H_2_ because of their target/filter and manufacturing similarity, and evaluated whether calibration data collected with one unit is valid when applied to another similarly manufactured unit. Likewise, we made direct comparisons between the H_3_ and GE units for the Mo/Mo and Mo/Rh combinations, when applicable.

**Figure 1:**
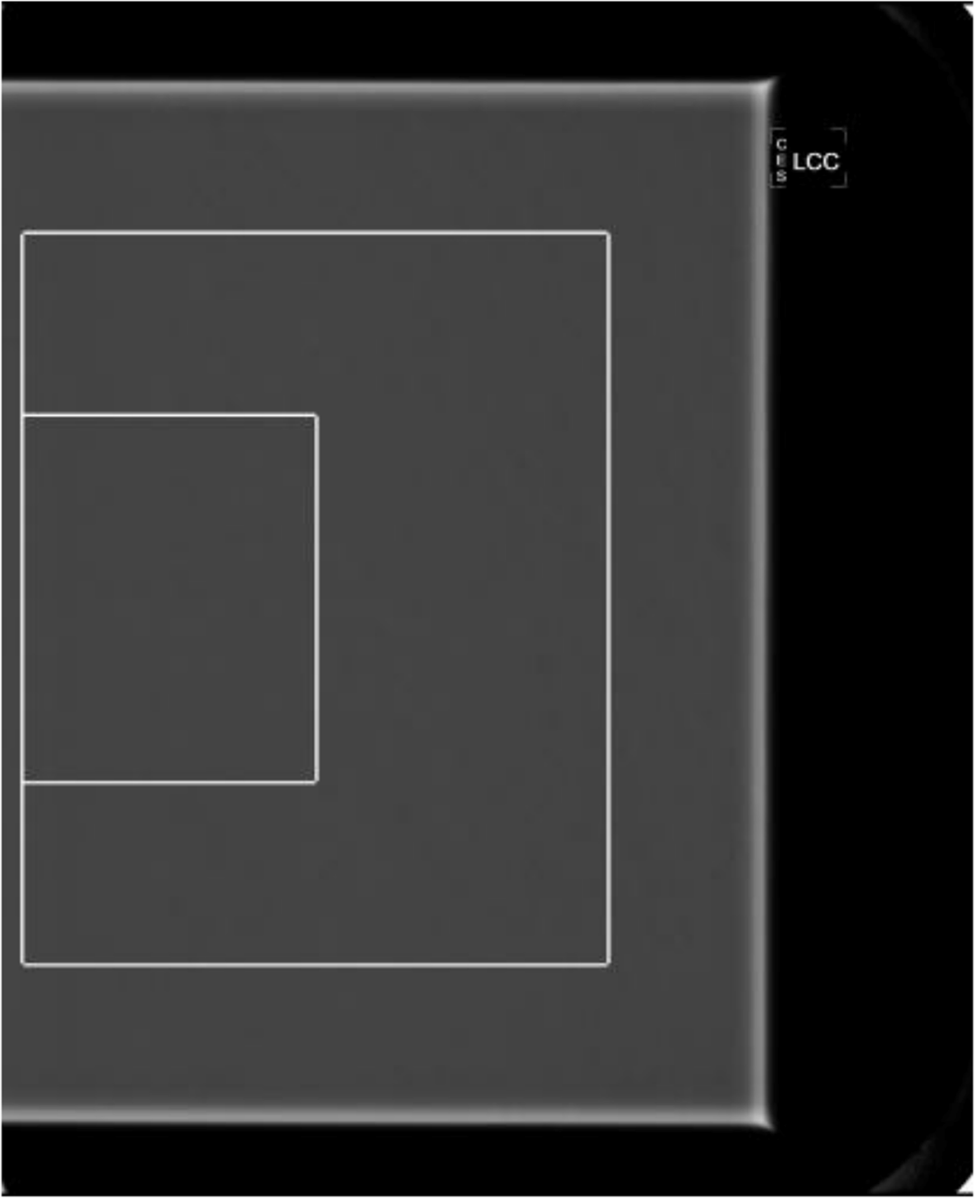
**Breast tissue equivalent phantom positioning, detector field of view, and regions of interest.** This shows the large field of view (largest rectangle) for the Hologic Selenia unit, the phantom (gray rectangle) placement on the detector (18 cm × 24 cm or approximately 2500 × 3400 pixels), and the regions of interest (ROIs) used for the analysis outlined with narrow light borders. The size of the large ROI is 2000 × 2500 pixels, and the size of the small ROI is 1000 × 1250 pixels.

The analysis was restricted to specific regions depending on the FFDM design and specific analysis endpoint. For the H_1_ H_2_ and H_3_ units, unless stated otherwise, the analysis was constrained to a large region of interest (ROI) specific to the large FOV. This ROI is defined as 2000 × 2500 pixels (14 cm × 17.5 cm), centered in the vertical direction with an horizontal offset of 75 pixels (not included) from the outside of the detector (i.e. parallel to the chest wall) or left border (LCC view). This restriction is to avoid stacked-phantom edge effects near the detector outer edge and possible flat field non-uniformity interference at regions far (interior) from the central detector area. For the FOV analysis and for images taken with the GE unit, the analysis was constrained to 1000 × 1250 pixel ROI with a 75 pixel offset (as above). The ROIs relative to the Hologic detector and the BTE phantom area are shown in Figure 1.

### Exposure response evaluation

We assessed the detector exposure and pixel value (pv) response relationships for the H_1_, H_2_, and H_3_ units for select kV settings for each target/filter combination, using the large FOV. The raw image pixel value (pv_raw_) response was modeled as a linear function of mAs by acquiring images without attenuation (i.e. open exposures of the detector). The mAs variable was sampled up to the point of detector saturation. The sample sets for each kV setting were analyzed with regression analysis and fitted to this form: <pv_raw_ > = m × x + b, where x is the system readout mAs quantity for each acquisition. The slope (m)_,_ intercept (b), coefficient of determination (R^2^), and standard error (SE) in the slope were used for evaluation purposes. The brackets indicate the mean pv_raw_ within the large ROI. We make the approximation that the system readout mAs value is a surrogate (proportional with) for the x-ray exposure at the detector, which is common practice. We made both intra-technology comparisons and comparisons with the GE exposure response, where applicable. Because H_1_ and H_2_ have the same target/filter combinations and H_3_ and GE have common combinations, the respective pairwise comparisons were included in the analysis. When making pairwise inter-unit slope comparisons for given kV, an important difference is defined as when the central value of m_i_ falls outside of this tolerance range: m_j_ ± 2 × SE_j_ or vice versa, where the index = 0 is reserved for the GE unit. Where appropriate, we compared the entire set of m_j_ across units with a t-test. Demonstrating that the response is linear has important implications in the BL calibration data collection requirements. When the linear approximation holds, the mAs sampling may be reduced to one sample in the BL dataset.

### Calibration dataset and characterization

The phantom imaging techniques and methods for constructing the BL calibration datasets (i.e. calibration curves) were described previously [21,22]. The same approach was applied in this report with some modification. Briefly, to construct the calibration curves for a given acquisition technique, two series of BTE phantoms were imaged to generate the respective glandular and adipose calibration curves for BL sampled heights defined as t_k_. Reference points derived from theses curves are used in the calibration application (discussed below). The phantom heights (total stacked heights) for a given calibration curve range from 2-7 cm depending on the acquisition technique, and were taken at 1 cm increments for convenience. To estimate the kV range, we selected the automated exposure control (auto-kV mode) and adjusted the compression paddle over a range of heights for fixed target/filter combinations. We estimated the W/Rh range is between 26-30 kV, and the W/Ag range is between 27-32 kV for the H_1_ and H_2_ systems. The same procedure was followed for Mo/Mo and Mo/Rh techniques for the H_3_ system giving 25-31 kV and 27-34 kV ranges, respectively. BL calibration datasets (H_1_, H_2_, H_3_ and GE units) were acquired with the same reference mAs setting defined as: x_r_ = 160 mAs. We selected a reference mAs value that does not cause detector saturation when imaging phantom configurations with smaller heights, in particular adipose phantoms, while providing sufficient signal when imaging phantoms with larger heights, in particular glandular phantoms, over the entire acquisition technique range considered, as discussed previously [22].

For both comparison and presentation purposes, we evaluated the calibration curves using linear regression methods without regard to calibration accuracy. We subdivided the large ROI (2000 × 2500 pixel region shown in Figure 1) into a grid consisting of 25 × 25 pixel smaller non-overlapping sub-regions defined as r_s_. This gives 80 × 96 = 7680 r_s_ sub-regions (for the large FOV). As above, t_k_ is the BL phantom height in cm with the index k designating a sampled height. For a given phantom configuration (fixed height and BTE type), we average the pixel values (i.e. <pv_raw_>) within r_s_ giving the mean exposure, E_l_(r_s_), at r_s_ and t_k._ For this report, the index, l, is reserved for the BTE type designation: l = a for adipose; and l = g for glandular. We divide E_l_(r_s_) by the reference mAs giving the relative mean exposure, RE_l_(r_s_) = E_l_(r_s_) / x_r_ (i.e. the reference x_r_ = 160 mAs) at each subdivision. We evaluate the natural logarithm of the relative mean exposure, LRE_l_(r_s_) = ln[RE_l_(r_s_)], as a function of increasing t_k_ giving a regional calibration curve; for reference, this defines logarithm of the relative exposure (LRE) domain, which holds at the pixel level as well. For inter-unit comparisons, we applied linear regression at each r_s_ for each BTE type resulting in a distribution for the slopes (μ_l_), logarithmic intercepts (LI_l_), and R^2^ values estimated by fitting the ordered pairs [t_k_, LRE_l_ (r_s_)] to this model

(1) $$\mathrm{LR}E_{l}=\mu_{l}\times t_{k}+LI_{l}.$$

When fitted to this form (t_k+1_ > t_k_), the magnitude of the slope can be interpreted as the effective x-ray attenuation coefficient (i.e. μ_g_ for glandular and μ_a_ for adipose tissue, cited as positive quantities in the tables and expressions) measured in cm^-1^ for a given kV and target/filter combination. The LI_l_ quantities are the respective intercepts, which are unit-less. We summarized these regression parameter distributions with the mean and mean standard error (SE). As above, we use the μ_l_ ± 2 × SE_l_ tolerance gauge for the inter-system pairwise comparisons. Where appropriate, we compared the entire set of effective x-ray attenuation coefficients across systems with a t-test for each BTE material. This sub-region analysis also gives a method for assessing the spatial uniformity of the calibration data.

### Calibration procedure

When calibrating an arbitrary image, the operation takes place in the LRE domain. In contrast to the calibration curve normalization that uses the reference mAs, the LRE for an arbitrary image (i.e. a prospective calibration application) is formed by normalizing either pv_raw_ or < pv_raw_ > by the acquisition system readout mAs defined as x before applying the natural logarithm given by: LRE = ln(pv_raw_/x). This normalization holds under certain conditions when the exposure response is linear. Similarly when the response is linear, two calibration points are required to calibrate an arbitrary image. These calibration points are derived from the BL curves and correspond to the theoretical pixel values in the LRE domain that would result when imaging materials that are (a) 100% glandular tissue = pv_g_, and (b) 100% adipose tissue = pv_a_ for a specific acquisition technique and height. For consistency with our past convention, we refer to the calibration domain as the percent glandular (PG) representation with values theoretically ranging from 0-100 PG units. This representation is analogous to a normalized x-ray attenuation coefficient representation, which is easily converted to total volume or average volumetric glandular metric by incorporating the compressed breast thickness (height) into the analysis [21]. The calibration mapping takes this form: PG_cal_ = M × LRE + B, where M and B are specific to a given kV, target/filter combination and height above the breast support surface; capitals are used to distinguish these parameters from the open detector exposure relationships. The LRE can be determined at the pixel level or sub-region level by using either the respective pixel value with the corresponding height or sub-region mean pixel value with corresponding mean height above the support surface.

For efficient prospective calibration applications, the BL calibration data must be stored. Therefore, we investigated two storage methods. The stored BL calibration data is then used in the specification of M and B. Both M and B are determined (fixed kV and target/filter) by considering the endpoints for a specific height t = t_0_. In the LRE domain, we set PG_cal_ = 100 when LRE = pv_g_, PG_cal_ = 0 when LRE = pv_a_ and solve for M and B: M = 100 × (pv_g_ – pv_a_)^-1^ and B = 50 – ½ M × (pv_g_ + pv_a_), giving one method for specifying M and B. In this specification approach, when t_0_ does not correspond exactly with a specific sample height from the BL, a cubic-spline interpolation was used to determine pv_g_ and pv_a_ at t_0_. The second method for specifying M and B expresses pv_a_ and pv_g_ as functions of the regressions parameters (μ_g_, μ_a_, LI_g_ and LI_a_ ) and t_0_ using Equation (1) by substituting t_k_ with t_0_: for example, pv_g_ ≈ − μ_g_ × t_0_ + LI_g_. In this case, the M and B specification and height interpolation are performed simultaneously; the validity of this approach relies on the agreement with Equation (1) and was the method developed previously for the GE unit [21,22]. With either specification method, the B relationship can be expressed in a simpler form to include only the pv_a_ term or the pv_g_ term, or the regression parameters from one of the calibration curves. We have included both measured terms (or all four regression parameters) to reduce variation in the event the curves or parameters carry dissimilar accuracy. We note, the 0–100 (PG units) calibration range is imposed by the development and it is not unique but follows intuition.

When applying the calibration, the large ROI within a given image is divided into 25 × 25 pixel sub-regions as above and the average of each sub-region is used in the calibration equation giving PG_cal_ = M × <LRE(r_s,_<t_0_>)> + B, where < t_0_ > is the mean height above the breast support surface about r_s_, resulting in a spatial distribution of calibrated values. The methods described in the Calibration dataset and characterization Section indicate the calibration curves, in the most general terms, are functions of position. For this report, we used the mean values of the calibration BL data taken over all r_s_ in the specification of M and B (both methods), removing the spatial dependency.

### Calibration accuracy evaluation

To evaluate the intra-machine calibration accuracy near the BL acquisition date (for the H_1_, H_2_, and H_3_ units), we imaged 4 cm composite phantoms comprised of a 2 cm adipose phantom stacked upon a 2 cm glandular phantom for the majority of kV settings and target/filter combinations. For a few of the larger kV acquisitions we used the same adipose and glandular ratio to construct 6 cm phantoms to avoid detector saturation. We refer to these composite phantoms as 50/50 mixtures. We also acquired 50/50 mixture images with three mAs settings to evaluate the impact of reference mAs normalization on the calibration accuracy: 120 mAs, 160 mAs (the reference) and 200 mAs (i.e. two additional samples for comparison purposes).

For the accuracy evaluation, we used the two methods outlined above for specifying M and B to select the optimal technique and make comparisons with our previous work. This evaluation was performed in four related steps. In step 1, we used the pv_a_ and pv_g_ determined with the BL dataset to calibrate 50/50 mixtures acquired with heights included in the BL; this should provide the best accuracy because no interpolation is required. In step 2, we calibrated the same mixtures used in step 1 with the regression parameter specification method; this does not permit a fair comparison with the first step because it includes interpolation but is required for the comparisons in the next two steps. To fully evaluate both interpolation methods, we also included additional 50/50 mixture acquisitions using the reference mAs (x_r_ = 160 mAs) with heights set at 4.2 cm, 4.4 cm and 6.4 cm, which were not included in the BL datasets (i.e. non-BL mixtures). In step 3, we used pv_a_ and pv_g_ derived from spline interpolation in the calibration of the non-BL mixtures, and in step 4 we used the regression parameters to calibrate the same non-BL mixtures. The comparison of step 1 with step 3 and comparison of step 2 with step 4 provides an intra-specification method evaluation by considering BL and non-BL height samples. The comparison of step 1 and 3 with step 2 and 4 provides a means for selecting the optimal interpolation method. From previous experience, we used an empirically derived tolerance of approximately ± 4 PG unit deviation from the ideal PG_cal_ = 50 for comparing calibration accuracy. For these comparisons, we acquired additional 50/50 mixtures using both BL heights (4 and 6 cm phantom heights) and non-BL heights. To minimize serial drift influences within the BL and non-BL comparison, we acquired both phantom series on the same day.

We performed two additional experiments to assess the calibration generality and accuracy. First, to evaluate whether calibration data acquired from one FFDM unit is applicable to another similar unit, we switched the BL calibration data and used BL_1_ (i.e. from H_1_) to calibrate 50/50 mixtures (with 160 mAs) acquired from H_2_ and vice versa, referred to as the cross-unit calibration analysis (findings discussed with those resulting from step 1). Secondly to evaluate FOV influences, we acquired 50/50 mixtures using the small FOV and performed calibration with the BL calibration data acquired with the large FOV for the H_1_, H_2_, and H_3_ units. To perform the small FOV analysis, a reduced ROI was used comprised of 1000 × 1250 pixels, outlined in Figure 1.

## Results

### Exposure response

**Figure 2:**
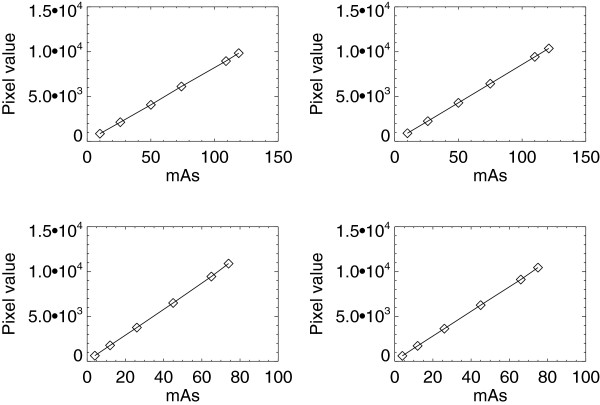
**Exposure response for the H**_**1 **_**and H**_**2 **_**units.** This shows exposure measured in milliampere seconds (mAs), the pixel value response, and fitted curves (solid) of the detector without attenuation for the H_1_ (left) and H_2_ (right) units. The measured values are averages represented by squares. The top plots show the W/Rh (target/filter) combinations acquired with 28 kV and the bottom plots show the W/Ag combinations acquired with 30 kV.

**Figure 3:**
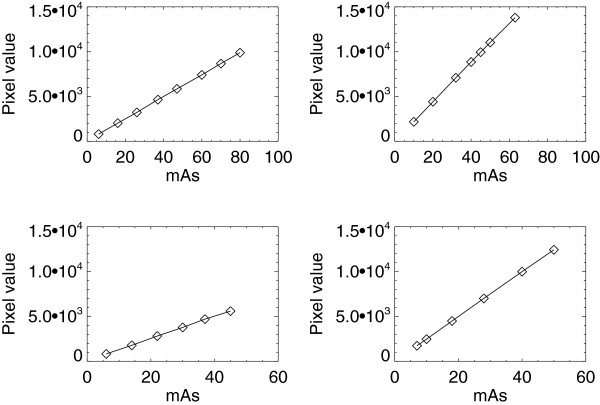
**Exposure response for the H**_**3 **_**and GE units.** This shows the exposure measured in milliampere seconds (mAs), the pixel value response, and fitted curves (solid) of the detector for the H_3_ (left) and GE (right) units. The measured values are averages represented by squares. The Mo/Mo (target/filter) combinations were acquired with 27 kV (top) and the Mo/Rh combinations (bottom) were acquired with 28 kV.

The open detector exposure relationships (pv and exposure response) for all systems are summarized in Table 1. Example plots are shown in Figure 2 for the similar H_1_ and H_2_ units. Plots for the H_3_ and GE units for common filter/target combinations are shown in Figure 3. The plots in both figures are representative of the linear response relationship for the four units. The R^2^ estimates (Table 1) are close to unity for all of the acquisition techniques considered, indicating the relationships are well approximated as linear for all units. Despite their design similarities, the response varies beyond our tolerance (i.e. m_j_ ± 2 × SE_j_) between the H_1_ and H_2_ units within kV settings. Although beyond the tolerance, the percent difference between m_1_ and m_2_ is within 3.3%-5.5%, whereas the intercepts show much larger variation. Comparing the set of m_1_ estimates with the set of m_2_ estimates (t-test) gave P > 0.96, indicating the exposure response does not differ significantly across similar systems. The pairwise responses also vary beyond the tolerance across the H_3_ and GE systems as expected for all observations. Although the exposure response quantities vary across all systems, the response linearity is a common characteristic across all units (H_1_, H_2_, H_3_, and GE). This common trait suggests the mAs sampling can be reduced to one sample for a given target/filter combination and kV setting (as evaluated below).

**Table 1:** Exposure and pixel value response analysis by target/filter and select kV combination

**H**_**1 **_**and H**_**2**_		**mAs**	**m**_**1 **_**(SE**_**1**_**)**	**b**_**1**_	**R**_**1**_^**2**^	**mAs**	**m**_**2 **_**(SE**_**2**_**)**	**b**_**2**_	**R**_**2**_^2^
W/Rh									
kV =	26	26 - 150	63.86 (0.15)	0.65	0.99	26 - 150	66.26 (0.15)	38.69	0.99
	28	10 - 120	82.37 (0.38)	3.06	0.99	10 - 120	85.24 (0.09)	43.44	0.99
	30	4 - 85	101.37 (0.66)	16.52	0.99	4 - 85	105.26 (0.17)	48.54	0.99
W/Ag									
kV =	28	4 - 85	113.52 (0.57)	19.31	0.99	4 - 85	108.44 (0.21)	52.47	0.99
	30	4 - 75	146.00 (1.04)	2.51	0.99	4 - 75	137.99 (0.51)	54.89	0.99
**H**_**3 **_**and GE**		**mAs**	**m**_**3 **_**(SE**_**3**_**)**	**b**_**3**_	**R**_**3**_^**2**^	**mAs**	**m**_**0 **_**(SE**_**0**_**)**	**b**_**0**_	**R**_**0**_^**2**^
Mo/Mo									
kV =	26	6 - 80	103.86 (0.30)	72.34	0.99	10 - 80	183.37 (0.89)	56.65	0.99
	27	6 - 80	122.71 (0.36)	72.29	0.99	10 - 65	219.59 (1.09)	29.72	0.99
Mo/Rh									
kV =	28	6 - 65	123.56 (0.60)	76.19	0.99	7 - 56	248.42 (0.78)	12.03	0.99
	29	6 - 65	142.01 (0.73)	69.55	0.99	10 - 45	288.13 (1.32)	3.09	0.99

H_1_: data acquired on 07/27/2012.

H_2_: data acquired on 07/20/2012.

H_3_: data acquired on 04/08/2013.

GE: data acquired on 10/13/2006.

GE: quantities referenced with the ′0′ subscript.

The detector exposure and pixel value response is modeled as a linear function of mAs for select kV settings: (1) the W/Rh and W/Ag target/filter combinations for the similar Hologic Selenia systems H_1_ and H_2_ (top); and (2) the Mo/Mo and Mo/Rh combinations for the third Hologic Selenia H_3_ system and General Electric Senographe 2000D (GE) system (bottom), designated by the subscript 0. The slopes (m_i_) and standard error (SE_i_), estimated as unit increase in pixel value per mAs, intercept (b_i_) measured in pixel value, and coefficient of determination (R_i_^2^) were derived from linear regression analysis. The mAs columns give the range of settings used for the acquisition techniques. The upper mAs value for a given technique was set close to where detector saturation occurs.

### Calibration datasets

**Figure 4:**
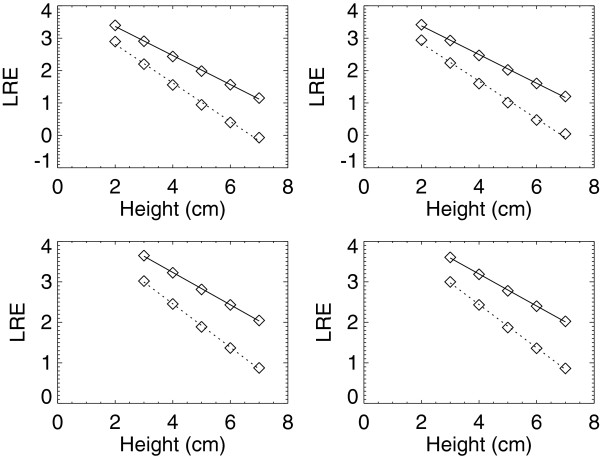
**Calibration curves for H**_**1 **_**and H**_**2 **_**units.** This shows the fitted adipose (solid) and glandular (dotted) calibration curves for the H_1_ (left-side plots) and H_2_ (right-side plots) units. The measured logarithmic relative response (LRE) quantities are averages represented by squares. The top plots show the W/Rh target/filter combinations acquired with 28 kV and the bottom plots show the W/Ag combinations acquired with 30 kV.

**Figure 5:**
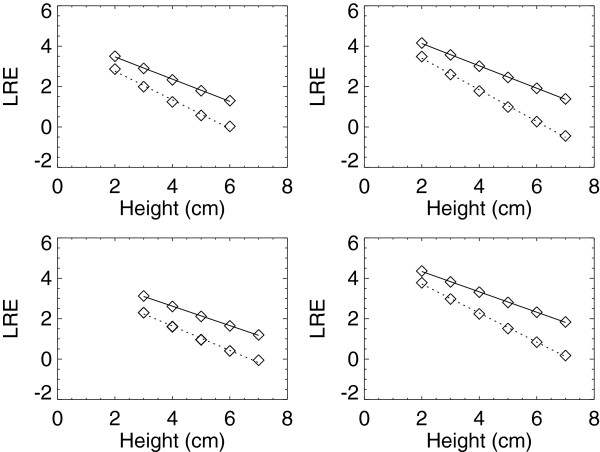
**Calibration curves for H**_**3 **_**and GE units.** This shows the fitted adipose (solid) and glandular (dotted) calibration curves for the H_3_ (left) and GE (right) units. The measured logarithmic relative response (LRE) quantities are averages represented by squares. The top plots show the Mo/Mo target/filter combinations acquired with 28 kV and the bottom plots show the Mo/Rh combinations acquired with 28 kV.

The effective attenuation coefficients (μ_l_) and logarithmic intercepts (LI_l_) for the H_1_ and H_2_ units are shown in Table 2 separated by the BTE type and FFDM unit. We have provided the absolute value of the slope from the regression analysis_,_ which is cited as μ_l_, and the corresponding SE_l_. Example calibration curve plots for these units fitted with regression analysis are shown in Figure 4. The R^2^ findings indicate the linear model fits well. The agreement of respective μ_l_ pair and SE vary. For example, the μ_a_ pairwise comparison for W/Rh combinations indicates there is close agreement for the 26–29 kV as gauged by the preset tolerance (μ_l_ ± 2 × SE_l_) with little variation at 26 kV and a maximum 2.3% variation at 30 kV, which is beyond the tolerance. The corresponding variations across the μ_g_ pairs show greater variation for the W/Rh combinations but are within the tolerance. The W/Ag glandular and adipose coefficients follow a similar trend and are within the similarity tolerance. Comparing the set of μ_a_ estimates for H_1_ with the corresponding set from H_2_ (t-test) gave P > 0.70. Similarly, comparing the μ_g_ set between H_1_ and H_2_ gave P > 0.45. These comparisons indicate the set of effective x-ray attenuation coefficients for a given BTE material does not differ significantly across similar systems. Because of the target/filter difference, no comparisons of the H_3_ and GE units with the H_1_ and H_2_ units are provided. The μ_l_, associated SE_l_, and LI_l_ for the H_3_ and GE units are shown in Table 3 for the Mo/Mo and Mo/Rh combinations, and example calibration curve plots fitted with regression analysis are shown in Figure 5. The R^2^ quantities indicate linearity is a common trait across these two different units. The pairwise attenuation coefficients are within magnitude agreement as are the LI_l_ quantities for these units but are not interchangeable or within the tolerance range when comparing the H_3_ and GE units. As above, comparing the μ_a_ set for H_3_ with the corresponding set for GE (t-test) gave P > 0.14, indicating the set of adipose x-ray attenuation coefficients is similar across systems that use different detector technologies. In contrast, the corresponding μ_g_ set comparison gave P < 0.0001, suggesting the attenuation coefficients for the glandular BTE material differ across these systems.

**Table 2:** **Baseline (BL) calibration dataset summary for the Hologic Selenia (H**_**1 **_**and H**_**2**_**) units**

**Target/filter and kV combination**		**Height range (cm)**	**Adipose BTE**			**Glandular BTE**		
			**μ**_**a **_**(SE)**	**LI**_**a**_	**R**^**2**^	**μ**_**g **_**(SE)**	**LI**_**g**_	**R**^**2**^
**H**_**1 **_**unit**								
W/Rh								
kV =	26	2 - 6	0.464 (0.008)	4.03	0.99	0.624 (0.022)	3.80	0.99
	27	2 - 6	0.461 (0.007)	4.17	0.99	0.620 (0.020)	3.93	0.99
	28	2 - 7	0.448 (0.008)	4.26	0.99	0.595 (0.020)	4.00	0.99
	29	3 - 7	0.432 (0.009)	4.31	0.99	0.560 (0.018)	3.94	0.99
	30	3 - 7	0.432 (0.007)	4.44	0.99	0.560 (0.016)	4.08	0.99
W/Ag								
kV =	27	3 - 7	0.412 (0.006)	4.40	0.99	0.539 (0.013)	4.09	0.99
	28	3 - 7	0.405 (0.006)	4.55	0.99	0.539 (0.013)	4.29	0.99
	29	3 - 7	0.403 (0.006)	4.71	0.99	0.538 (0.010)	4.45	0.99
	30	3 - 7	0.400 (0.005)	4.83	0.99	0.537 (0.010)	4.61	0.99
	31^a^	3 - 7	0.406 (0.006)	5.04	0.99	0.533 (0.011)	4.76	0.99
	32^b^	4 - 7	0.392 (0.004)	5.08	0.99	0.512 (0.011)	4.79	0.99
**H**_**2 **_**unit**								
W/Rh								
kV =	26	2 - 6	0.464 (0.008)	4.06	0.99	0.616 (0.026)	3.82	0.99
	27	2 - 6	0.462 (0.008)	4.21	0.99	0.621 (0.019)	3.97	0.99
	28	2 - 7	0.442 (0.008)	4.27	0.99	0.581 (0.023)	4.00	0.99
	29	3 - 7	0.431 (0.006)	4.35	0.99	0.536 (0.026)	3.90	0.99
	30	3 - 7	0.422 (0.007)	4.42	0.99	0.540 (0.020)	4.06	0.99
W/Ag								
kV =	27	3 - 7	0.404 (0.005)	4.34	0.99	0.518 (0.019)	4.01	0.99
	28	3 - 7	0.401 (0.004)	4.49	0.99	0.522 (0.015)	4.23	0.99
	29	3 - 7	0.402 (0.004)	4.66	0.99	0.520 (0.014)	4.37	0.99
	30	3 - 7	0.395 (0.006)	4.77	0.99	0.534 (0.009)	4.58	0.99
	31	3 - 7	0.395 (0.005)	4.91	0.99	0.524 (0.009)	4.67	0.99
	32^b^	4 - 7	0.388 (0.005)	5.01	0.99	0.506 (0.011)	4.72	0.99

H_1_: W/Rh data acquired on 07/06/2012; W/Ag 27-30 kV data acquired on 07/06/2012.

H_2_: W/Rh data acquired on 07/10/2012; W/Ag 27-31 kV data acquired on 07/10/2012.

a: data acquired on 07/09/2012.

b: data acquired on 07/17/2012.

The effective x-ray attenuation coefficients, μ_a_ and μ_g_, logarithmic intercepts, LI_a_ and LI_g_, and coefficient of determination (R^2^) were derived with regression analysis for the similar Hologic (H_1_ and H_2_ ) units for the W/Rh and W/Ag target/filter combinations are provided as mean values. The mean μ_l_ and the associated mean standard error (SE_l_) are cited. The x-ray tube voltage (kV) and phantom height ranges are provided. For each kV setting, the images were acquired by incrementing the phantom heights from the lower range to the upper range in 1 cm increments. The absolute value of the slope is equivalent to the effective x-ray attenuation coefficient for each acquisition technique and BTE material.

**Table 3:** Baseline (BL) calibration data summary for two different FFDM technologies

**Target/filter and kV combination**		**Height range (cm)**	**Adipose BTE**			**Glandular BTE**		
			**μ**_**a **_**(SE)**	**LI**_**a**_	**R**^**2**^	**μ**_**g **_**(SE)**	**LI**_**g**_	**R**^**2**^
**H**_**3 **_**unit**								
Mo/Mo								
kV =	25	2 - 6	0.561 (0.014)	4.18	0.99	0.705 (0.047)	3.69	0.99
	26	2 - 6	0.557 (0.011)	4.40	0.99	0.706 (0.043)	3.92	0.99
	27	2 - 6	0.551 (0.012)	4.57	0.99	0.709 (0.036)	4.18	0.99
	28	2 - 7	0.526 (0.012)	4.70	0.99	0.639 (0.039)	4.15	0.99
	29	3 - 7	0.495 (0.010)	4.74	0.99	0.586 (0.029)	4.09	0.99
	30	3 - 7	0.486 (0.009)	4.89	0.99	0.568 (0.030)	4.22	0.99
	31	3 - 7	0.470 (0.011)	4.99	0.99	0.560 (0.026)	4.42	0.99
Mo/Rh								
kV =	27	3 - 7	0.476 (0.009)	4.35	0.99	0.586 (0.031)	3.80	0.99
	28	3 - 7	0.482 (0.008)	4.55	0.99	0.592 (0.026)	4.01	0.99
	29	3 - 7	0.475 (0.008)	4.67	0.99	0.595 (0.024)	4.20	0.99
	30	3 - 7	0.471 (0.008)	4.83	0.99	0.585 (0.025)	4.32	0.99
	31	3 - 7	0.463 (0.008)	4.93	0.99	0.582 (0.021)	4.49	0.99
	32	3 - 7	0.459 (0.009)	5.07	0.99	0.564 (0.022)	4.57	0.99
	33	3 - 7	0.445 (0.008)	5.14	0.99	0.554 (0.021)	4.71	0.99
	34	3 - 7	0.443 (0.008)	5.28	0.99	0.533 (0.020)	4.77	0.99
**GE unit**								
Mo/Mo								
kV =	25*	2 - 6	0.584 (0.004)	4.93	0.99	0.861 (0.014)	4.74	0.99
	26	2 - 6	0.572 (0.005)	5.09	0.99	0.833 (0.016)	4.89	0.99
	27	2 - 6	0.560 (0.005)	5.26	0.99	0.805 (0.017)	5.04	0.99
	28*	2 - 7	0.548 (0.005)	5.43	0.99	0.777 (0.019)	5.19	0.99
	29*	3 - 7	0.536 (0.006)	5.60	0.99	0.749 (0.021)	5.34	0.99
	30*	3 - 7	0.524 (0.006)	5.77	0.99	0.721 (0.023)	5.49	0.99
Mo/Rh								
kV =	27*	3 - 7	*0.512 (0.005)	5.21	0.99	0.736 (0.011)	5.03	0.99
	28	3 - 7	0.504 (0.005)	5.34	0.99	0.718 (0.013)	5.15	0.99
	29	3 - 7	0.495 (0.004)	5.47	0.99	0.700 (0.014)	5.27	0.99
	30*	3 - 7	*0.487 (0.004)	5.60	0.99	0.682 (0.016)	5.39	0.99
	31*	3 - 7	*0.479 (0.004)	5.74	0.99	0.665 (0.017)	5.51	0.99

H_3_: data acquired on 04/08/2013.

GE: data acquired on 10/13/2006.

* Interpolated values.

The effective x-ray attenuation coefficients, μ_a_ and μ_g_, logarithmic intercepts LI_a_ and LI_g_, and coefficient of determination (R^2^), derived with regression analysis, for the Hologic (H_3_) and the General Electric Senographe 2000D (GE) FFDM systems for the Mo/Mo and Mo/Rh target/filter combinations are provided as mean values. The mean μ_l_ and the associated mean standard error (SE_l_) are cited. The x-ray tube voltage (kV) and phantom height (thickness) ranges are provided. For each kV sample, images were acquired by incrementing the phantom heights from the lower range limit to the upper range limit in 1 cm increments. The absolute value of the slope, as cited, is equivalent to the effective attenuation coefficient for a given BTE material.

### Calibration accuracy

For the BL calibration accuracy evaluation, the spline specification method findings (step 1) are presented in this section because the M and B are specified by the calibration points at t_k_, which are special cases. For the most part as shown in Table 4, the within-unit accuracy for the H_1_ and H_2_ units is within ± 4 PG units of the ideal value (i.e. PG_cal_ = 50). However, there is greater variation for W/Ag acquisitions in the larger kV settings. This may be because the H_1_ calibration data for these samples was acquired on a different date than the rest of the respective BL dataset. The within-unit W/Ag accuracy for the most part is similar to the intra-system accuracy, whereas the accuracy for the W/Rh shows greater variation from the ideal value. The accuracy for the examples taken with non-reference mAs settings are similar to those obtained with the 160 mAs reference, showing the validity of the LRE normalization. The cross-unit calibration findings, provided in the right side of Table 4 for H_1_ and H_2_ units, show a trend beyond our tolerance gauge of ± 4 PG. These findings suggest that the calibration data in general is specific to the unit, even though they are *identical*. In addition to the x-ray attenuation coefficient differences, another source of variation stems from the LIs, which may vary due to the inter-system exposure response differences (Table 1). The accuracy evaluation for H_3_ is shown in Table 5 using the same format (without cross-unit calibration). The Mo/Mo and Mo/Rh accuracies marginally exceed the tolerance gauge but are similar across the mAs range. Because we do not have similar experiments performed with the GE unit, direct comparisons are not possible. However, in general, the accuracies obtained with H_3_, as well as the H_1_ and H_2_ units, are similar to those obtained with GE previously [22]. The accuracies shown in Tables 4 and 5 with the respective standard deviations (SDs) indicate that spatial non-uniformity has a minimal influence.

**Table 4:** **Calibration accuracy for the H**_**1 **_**and H**_**2 **_**units**

**Target/filter and kV combination**		**Within-unit calibration**						**Cross-unit calibration**					
		**H**_**1**_			**H**_**2**_			**H**_**1 **_**images calibrated with H**_**2 **_**data**			**H**_**2 **_**images calibrated with H**_**1 **_**data**		
		**120mAs**	**160mAs**_**r**_	**200mAs**	**120mAs**	**160mAs**_**r**_	**200mAs**	**120mAs**	**160mAs**_**r**_	**200mAs**	**120mAs**	**160mAs**_**r**_	**200mAs**
W/Rh													
kV =	26	50.6(0.44)	51.9(0.47)	54.0(0.47)	48.8(0.48)	51.6(0.44)	52.9(0.40)	55.7(0.44)	57.1(0.47)	59.2(0.47)	43.6(0.48)	46.5(0.44)	47.8(0.40)
	27	50.2(0.44)	51.8(0.46)	53.5(0.44)	49.2(0.43)	50.6(0.43)	52.3(0.42)	54.2(0.44)	55.9(0.46)	57.6(0.44)	45.2(0.43)	46.5(0.43)	48.2(0.42)
	28	50.8(0.46)	52.3(0.46)	53.5(0.46)	50.8(0.37)	51.8(0.33)	53.2(0.35)	55.5(0.47)	57.0(0.47)	58.2(0.46)	46.2(0.36)	47.2(0.33)	48.5(0.35)
	29	50.3(0.47)	51.4(0.50)	52.7(0.48)	51.2(0.31)	52.6(0.30)	53.3(0.31)	55.9(0.47)	57.0(0.50)	58.3(0.49)	45.7(0.31)	47.1(0.29)	47.8(0.31)
	30	50.9(0.61)	52.1(0.63)	53.0(0.62)	51.5(0.39)	52.5(0.40)	53.1(0.42)	55.8(0.62)	57.0(0.65)	57.9(0.63)	46.7(0.38)	47.8(0.39)	48.3(0.41)
W/Ag													
kV =	27	51.0(0.77)	52.4(0.78)	52.9(0.80)	52.3(0.43)	53.3(0.44)	54.0(0.44)	50.9(0.80)	52.4(0.81)	52.9(0.83)	52.3(0.42)	53.3(0.42)	53.9(0.43)
	28	51.1(0.90)	52.1(0.92)	52.8(0.93)	52.2(0.39)	53.5(0.41)	52.5(0.43)	50.1(0.92)	51.1(0.94)	51.8(0.95)	53.1(0.38)	54.4(0.39)	53.5(0.42)
	29	50.8(0.94)	52.1(0.96)	52.2(0.97)	51.5(0.40)	52.8(0.41)	52.9(0.40)	49.5(0.99)	50.7(0.98)	50.9(0.99)	52.8(0.39)	54.1(0.40)	54.2(0.39)
	30	51.5(0.98)	52.5(0.99)	52.6(1.00)	52.5(0.44)	53.0(0.44)	53.4(0.45)	48.9(0.99)	50.0(1.00)	50.0(1.01)	55.0(0.43)	55.6(0.44)	55.9(0.45)
	31	55.9(0.91)	56.7(0.90)	56.8(0.93)	52.3(0.51)	53.2(0.51)	53.6(0.52)	48.3(0.94)	49.1(0.93)	49.3(0.97)	59.7(0.49)	60.6(0.49)	61.0(0.50)
	32	54.0(0.97)	54.8(0.97)	55.9(1.00)	54.0(0.64)	54.6(0.64)	55.8(0.65)	51.2(0.98)	52.1(0.97)	53.2(1.00)	56.7(0.63)	57.3(0.64)	58.5(0.65)

Within-unit Calibration: 50/50 mixtures acquired and calibrated with the same FFDM unit.

Cross-unit Calibration: 50/50 mixtures acquired with each FFDM unit were calibrated with the other unit's calibration data.

H_1_: phantom data acquired on 07/27/2012 and calibration data on 07/06/2012.

H_2_: phantom data acquired on 07/20/2012 and calibration data on 07/10/2012.

This table summarizes the results from the within-unit calibration (left side) and cross-unit calibration (right side) for H_1_ and H_2_ units. For each target/filter combination and kV setting, the 50/50 mixture phantom images were acquired with three mAs settings including the reference denoted by mAs_r_. The mean and the standard deviation (SD) of the respective distributions are provided in PG units.

**Table 5:** **Within-unit calibration accuracy for the H**_**3**_

**Target/filter and kV combination**		**Within-unit Calibration: H**_**3**_		
		**120mAs**	**160mAs**_**r**_	**200mAs**
Mo/Mo				
kV =	25	49.4(0.53)	54.5(0.49)	56.1(0.48)
	26	50.3(0.47)	53.7(0.40)	55.3(0.41)
	27	51.6(0.36)	54.6(0.34)	55.1(0.35)
	28	52.2(0.24)	54.6(0.23)	55.0(0.25)
	29	53.4(0.16)	55.4(0.17)	55.6(0.19)
	30	52.8(0.16)	55.1(0.20)	55.3(0.21)
	31	53.3(0.27)	55.7(0.32)	55.7(0.32)
Mo/Rh				
kV =	27	51.8(1.44)	53.6(1.52)	54.2(1.51)
	28	52.6(1.45)	53.8(1.52)	54.0(1.53)
	29	52.8(1.44)	54.0(1.52)	54.4(1.51)
	30	53.7(1.35)	54.5(1.42)	54.8(1.42)
	31	53.0(1.30)	54.6(1.37)	54.7(1.35)
	32	53.3(1.16)	54.6(1.24)	54.2(1.19)
	33	54.3(1.09)	55.0(1.15)	55.0(1.14)
	34	55.6(0.94)	55.6(1.00)	55.8(0.98)

Within-unit Calibration: 50/50 mixtures acquired and calibrated with the same FFDM unit.

For each target/filter and kV setting, the 50/50 mixture phantom images were acquired with three mAs settings, including the reference denoted by mAs_r_. The mean and the standard deviation (SD) of the respective distributions are provided in PG units.

Table 6 shows the calibration generated with linear regression parameter specification method (i.e. step 2) for the H_1_ and H_2_ units. For the 160 mAs reference examples, the accuracy for 5 of the 11 acquisition techniques was outside of the ± 4 PG tolerance for the H_1_ unit. Similarly, the calibration was beyond the tolerance for 6 of the 11 acquisition techniques for H_2_. For the H_3_ unit, the accuracy was beyond the tolerance for all 15 acquisition techniques and exceeded +7 PG for 9 of these techniques (data not shown to limit the presentation). The accuracy for non-reference mAs examples follows a similar accuracy trend. The accuracies in Table 6 should be compared with respective findings in Table 4 (left side).

**Table 6:** **Calibration accuracy for the H**_**1 **_**and H**_**2 **_**units using the regression parameters**

**Target/filter and kV combination**		**Within-unit calibration**					
		**H**_**1**_			**H**_**2**_		
		**120mAs**	**160mAs**_**r**_	**200mAs**	**120mAs**	**160mAs**_**r**_	**200mAs**
W/Rh							
kV =	26	55.2 (0.46)	56.6 (0.49)	58.8 (0.48)	53.4 (0.50)	56.3 (0.46)	57.7 (0.42)
	27	54.0 (0.45)	55.7 (0.47)	57.4 (0.45)	53.2 (0.44)	54.6 (0.44)	56.3 (0.43)
	28	56.1 (0.48)	57.7 (0.48)	58.9 (0.48)	56.8 (0.39)	57.9 (0.35)	59.4 (0.37)
	29	51.8 (0.47)	53.0 (0.50)	54.2 (0.48)	52.7 (0.32)	54.1 (0.30)	54.8 (0.32)
	30	52.4 (0.61)	53.6 (0.64)	54.5 (0.62)	52.8 (0.39)	53.9 (0.40)	54.5 (0.43)
W/Ag							
kV =	27	52.0 (0.78)	53.4 (0.79)	53.9 (0.81)	53.2 (0.44)	54.2 (0.44)	55.0 (0.44)
	28	52.3 (0.89)	53.3 (0.91)	54.0 (0.93)	53.0 (0.40)	54.4 (0.41)	53.4 (0.43)
	29	52.0 (0.94)	53.2 (0.96)	53.3 (0.97)	52.3 (0.40)	53.6 (0.41)	53.8 (0.41)
	30	52.2 (0.98)	53.2 (0.99)	53.3 (1.00)	53.4 (0.44)	54.0 (0.45)	54.4 (0.45)
	31	57.1 (0.91)	57.9 (0.90)	58.0 (0.93)	53.2 (0.51)	54.1 (0.51)	54.5 (0.52)
	32	54.8 (0.97)	55.6 (0.97)	56.8 (1.00)	54.9 (0.64)	55.5 (0.65)	56.7 (0.66)

Within-unit Calibration: 50/50 mixtures acquired and calibrated with a same FFDM unit.

H_1_: Phantom data acquired on 07/27/2012 and calibration data on 07/06/2012.

H_2_: Phantom data acquired on 07/20/2012 and calibration data on 07/10/2012.

This table gives the calibration findings for the two similar H_1_ and H_2_ FFDM systems determined with the regression parameters. The mean and the standard deviation (SD) of the respective distributions are provided in PG units. For each target/filter and kV setting, the 50/50 mixture phantom images were acquired with three mAs settings including the reference denoted as mAs_r_.

The cubic-spline height interpolation findings for the H_1_, H_2_, and H_3_ systems are shown in Table 7 for the non-BL evaluation (step 3). When comparing either within or across the H_1_ and H_2_ systems, the findings show that non-BL height accuracy is within the ± 4 PG tolerance for all but one acquisition technique indicating similarity across systems and the validity of the spline interpolation. The right portion of Table 7 shows the H_3_ evaluation for the Mo/Mo and Mo/Rh examples. Although the calibration accuracies are marginally above the tolerance for both the BL and non-BL heights, the accuracies are similar to those shown in Table 5, again demonstrating the validity of the spline interpolation technique. The regression parameter interpolation findings for the non-BL evaluation are shown in Table 8 (step 4). The accuracies for the non-BL from H_1_ are within the tolerance, whereas the majority of the H_2_ accuracies are beyond the tolerance. Although the H_3_ accuracy is in agreement with its related findings (Table 5), the BL accuracies are beyond the tolerance, and the non-BL calibration quantities deviate beyond the BL quantities. In summary, interpolation with the regression parameter method is inferior to the spline method when considering the H_1_, H_2_, and H_3_ units in combination. We note, the H_3_ findings for both BL and non-BL examples are consistently beyond the tolerance in contrast with H_1_ and H_2_ findings. At this time, we cannot account for this discrepancy.

**Table 7:** Calibration accuracy using the cubic-spline height interpolation

**Target/filter and kV combination**			**Within-unit calibration: H**_**1**_		**Within-unit calibration: H**_**2**_		**Target/filter and kV combination**			**Within-unit calibration: H**_**3**_	
		**Non-BL height (cm)**	**BL**	**Non-BL**	**BL**	**Non-BL**			**Non-BL height (cm)**	**BL**	**Non-BL**
W/Rh							Mo/Mo				
kV =	25	--	--	--	--	--	kV =	25	4.2	54.5 (0.49)	54.9 (0.84)
	26	4.2	46.5 (0.36)	48.6 (0.67)	51.1 (0.41)	52.8 (0.76)		26	4.4	53.7 (0.40)	54.5 (0.47)
	27	4.4	48.6 (0.67)	48.8 (0.42)	51.4 (0.43)	50.9 (0.82)		27	4.2	54.6 (0.34)	54.7 (0.70)
	28	4.2	48.4 (0.39)	49.6 (0.65)	51.8 (0.68)	53.2 (0.91)		28	4.4	54.6 (0.23)	54.7 (0.32)
	29	4.4	47.8 (0.49)	48.3 (0.53)	51.3 (0.56)	52.9 (0.63)		29	4.2	55.4 (0.17)	55.2 (0.56)
	30	4.2	48.2 (0.70)	48.5 (0.80)	51.9 (0.51)	51.7 (0.88)		30	4.4	55.1 (0.20)	54.8 (0.31)
	31	--	--	--	--	--		31	4.2	55.7 (0.32)	55.9 (0.52)
W/Ag							Mo/Rh				
kV =	27	4.2	46.5 (0.73)	47.8 (0.91)	52.0 (0.54)	53.5 (0.89)	kV =	27	4.2	53.6 (1.52)	53.9 (1.68)
	28	4.4	47.3 (0.85)	48.0 (0.96)	52.1 (0.52)	51.8 (0.65)		28	4.4	53.8 (1.52)	54.2 (1.38)
	29	4.2	47.5 (0.90)	48.4 (1.10)	50.9 (0.49)	52.4 (0.73)		29	4.2	54.0 (1.52)	54.2 (1.68)
	30	4.4	47.8 (0.95)	48.0 (0.99)	51.6 (0.52)	51.5 (0.54)		30	4.4	54.5 (1.42)	54.3 (1.23)
	31	4.2	52.4 (0.86)	52.8 (1.13)	52.0 (0.50)	52.0 (0.56)		31	4.2	54.6 (1.37)	54.7 (1.52)
	32	6.4	51.9 (0.75)	52.4 (0.78)	53.6 (0.57)	54.5 (0.61)		32	4.4	54.6 (1.24)	54.1 (0.98)
	33	--	--	--	--	--		33	4.2	55.0 (1.15)	55.0 (1.29)
	34	--	--	--	--	--		34	4.4	55.6 (1.00)	52.9 (0.76)

Within-unit Calibration: 50/50 mixtures acquired and calibrated with a same FFDM unit.

H_1_: data acquired on 08/10/2012.

H_2_: data acquired on 08/06/2012.

H_3_: data acquired on 04/08/2013.

32 kV W/Ag BL acquired at 6.0 cm.

50/50 mixture phantom images were acquired with the 160 mAs reference setting for heights (right most column) not considered in the baseline sampling (Non-BL) and calibrated to evaluate the cubic-spline height interpolation. The findings are provided as the mean and the standard deviation (parenthetically) for the respective distributions in PG units. The calibration findings are also provided for 50/50 mixture phantom images acquired with the standard BL heights close to (4 cm or 6 cm) the non-BL heights, which were acquired on the same day for comparison purposes.

**Table 8:** Calibration Accuracy using the regression parameters

**Target/filter and kV combination**			**Within-unit calibration: H**_**1**_		**Within-unit calibration: H**_**2**_		**Target/filter and kV combination**			**Within-unit calibration: H**_**3**_	
		**Non-BL height (cm)**	**BL**	**Non-BL**	**BL**	**Non-BL**			**Non-BL height (cm)**	**BL**	**Non-BL**
W/Rh							Mo/Mo				
kV =	25	--	--	--	--	--	kV =	25	4.2	62.8 (0.53)	63.3 (0.92)
	26	4.2	50.9 (0.37)	53.1 (0.70)	55.8 (0.43)	57.8 (0.80)		26	4.4	60.8 (0.43)	61.5 (0.51)
	27	4.4	51.6 (0.36)	52.7 (0.44)	55.4 (0.43)	54.8 (0.84)		27	4.2	61.2 (0.36)	61.2 (0.74)
	28	4.2	53.6 (0.40)	55.2 (0.68)	57.9 (0.72)	59.8 (0.97)		28	4.4	63.5 (0.25)	64.5 (0.36)
	29	4.4	49.3 (0.49)	51.4 (0.54)	52.7 (0.57)	55.7 (0.65)		29	4.2	58.0 (0.18)	59.2 (0.58)
	30	4.2	49.6 (0.71)	50.8 (0.81)	53.2 (0.52)	54.0 (0.90)		30	4.4	57.7 (0.20)	59.7 (0.33)
	31	--	--	--	--	--		31	4.2	58.2 (0.33)	59.7 (0.53)
W/Ag							Mo/Rh				
kV =	27	4.2	47.5 (0.73)	49.5 (0.93)	52.9 (0.54)	55.3 (0.90)	kV =	27	4.2	55.8 (1.55)	57.5 (1.74)
	28	4.4	48.5 (0.84)	50.4 (0.97)	53.0 (0.52)	54.3 (0.66)		28	4.4	55.8 (1.54)	58.1 (1.43)
	29	4.2	48.6 (0.90)	50.2 (1.11)	51.7 (0.49)	54.0 (0.74)		29	4.2	55.7 (1.55)	57.0 (1.73)
	30	4.4	48.5 (0.94)	50.0 (1.00)	52.6 (0.52)	53.6 (0.55)		30	4.4	56.3 (1.45)	58.1 (1.28)
	31	4.2	53.6 (0.87)	54.8 (1.14)	52.9 (0.50)	53.5 (0.56)		31	4.2	56.9 (1.39)	58.0 (1.56)
	32	6.4	52.7 (0.76)	52.5 (0.78)	54.5 (0.57)	54.6 (0.62)		32	4.4	57.1 (1.26)	58.4 (1.01)
	33	--	--	--	--	--		33	4.2	57.4 (1.18)	58.5 (1.33)
	34	--	--	--	--	--		34	4.4	57.6 (1.02)	56.9 (0.79)

Within-unit Calibration: 50/50 mixtures acquired and calibrated with a same FFDM unit.

H_1_: data acquired on 08/10/2012.

H_2_: data acquired on 08/06/2012.

H_3_: data acquired on 04/08/2013.

32 kV W/Ag BL acquired at 6.0 cm.

50/50 mixture phantom images were acquired with the 160 mAs reference setting for heights (right most column) not considered in the BL sampling (Non-BL) and calibrated to assess the regression parameter height interpolation. The findings are provided as the mean and the standard deviation (parenthetically) for the respective distributions in PG units. The calibration findings are also provided for 50/50 images acquired with the standard BL heights close to (4 cm or 6 cm) the non-BL heights, which were acquired on the same day for comparison proposes.

In the final analysis, we assessed the potential influence of the system FOV for the H_1_, H_2_ and H_3_ units (cubic-spline approach). Table 9 shows the findings when applying the calibration data acquired with the large FOV to 50/50 mixtures taken with the small FOV. For comparison, 50/50 mixtures acquired with the large FOV were also calibrated; both sets of images were acquired on the same day to minimize serial drift influences. Considering the large FOV findings as the standards, the respective small FOV calibration accuracy is well within ± 4 PG tolerance, demonstrating the FOV change has little influence.

**Table 9:** Calibration accuracy for images acquired with the small FOV calibrated with data acquired with the large FOV

**Target/filter and kV combination**		**H**_**1**_		**H**_**2**_		**Target/filter and kV combination**		**H**_**3**_	
		**Small FOV**	**Large FOV**	**Small FOV**	**Large FOV**			**Small FOV**	**Large FOV**
W/Rh						Mo/Mo			
kV =	25	--	--	--	--	kV =	25	53.0 (0.37)	54.5 (0.49)
	26	54.5 (0.38)	51.9 (0.47)	51.8 (0.28)	51.6 (0.44)		26	52.3 (0.30)	53.7 (0.40)
	27	53.0 (0.38)	51.8 (0.46)	48.2 (0.34)	50.6 (0.43)		27	53.1 (0.24)	54.6 (0.34)
	28	53.2 (0.44)	52.3 (0.46)	51.5 (0.42)	50.6 (0.43)		28	53.3 (0.17)	54.6 (0.23)
	29	52.5 (0.50)	51.4 (0.50)	51.5 (0.55)	51.8 (0.33)		29	54.2 (0.14)	55.4 (0.17)
	30	53.0 (0.64)	52.1 (0.63)	51.6 (0.72)	52.6 (0.30)		30	54.1 (0.17)	55.1 (0.20)
	31	--	--	--	--		31	54.7 (0.25)	55.7 (0.32)
W/Ag						Mo/Rh			
kV =	27	52.2 (0.39)	52.4 (0.78)	52.7 (0.70)	53.3 (0.44)	kV =	27	51.2 (1.15)	53.6 (1.52)
	28	52.1 (0.42)	52.1 (0.92)	52.2 (0.72)	53.5 (0.41)		28	51.7 (1.15)	53.8 (1.52)
	29	50.9 (0.42)	52.1 (0.96)	52.1 (0.75)	52.8 (0.41)		29	51.6 (1.15)	54.0 (1.52)
	30	52.1 (0.43)	52.5 (0.99)	51.9 (0.86)	53.0 (0.44)		30	52.3 (1.08)	54.5 (1.42)
	31	56.3 (0.41)	56.7 (0.90)	52.2 (0.96)	53.2 (0.51)		31	52.3 (1.08)	54.6 (1.37)
	32	54.9 (0.38)	54.8 (0.97)	54.5 (1.00)	54.6 (0.64)		32	52.7 (1.01)	54.6 (1.24)
	33	--	--	--	--		33	53.3 (0.99)	55.0 (1.15)
	34	--	--	--	--		34	53.8 (0.91)	55.6 (1.00)

H_1_: data acquired on 07/27/2012.

H_2_: data acquired on 07/20/2012.

H_3_: data acquired on 04/08/2012.

50/50 mixture phantom images were acquired with small field of view (FOV) using the standard BL heights and the 160 mAs reference setting for and calibrated with data acquired with the large FOV for the H_1_, H_2_, and H_3_ FFDM units (Hologic units). The large FOV calibration results are provided for reference. The findings are provided as the mean and the standard deviation (parenthetically) of the respective distributions in PG units.

## Discussion

A calibration system for Hologic Selenia FFDM units was established upon our previous work [21,22] using a different FFDM technology. The findings demonstrate the generality of our approach. There are both important similarities and differences when comparing the inter-FFDM technology calibration requirements. The mAs normalization was similar across the two technologies and is dependent in part upon the linearity of the pixel value and exposure relationship and the validity of ignoring the intercept term (i.e. assuming the relationship is proportional in addition to linear). The findings suggest that at a minimum, one reference mAs sample may be sufficient for generating calibration curves in agreement with our previous findings. We showed that the calibration data could be acquired with the large FOV only without impacting the calibration accuracy for images acquired with the small FOV. The ability to use a single reference mAs and FOV results in a substantial reduction in data collection required to establish the BL calibration datasets. Although the calibration curves were well approximated as linear for the systems evaluated in this report, we required a cubic-spline height interpolation for the H_1_, H_2_, and H_3_ units. This spline interpolation requirement is in contrast with our previous work, where the effective x-ray attenuation coefficients and logarithmic intercepts (i.e. regression parameters) were stored and then used for generating both the height interpolation and calibration points. Consistent with our findings from similar GE systems [22], each similarly-manufactured Hologic system (i.e. H_1_ and H_2_) requires its own BL calibration dataset to maintain acceptable calibration accuracy.

There are several limitations with this work. The data was collected over a period of approximately 35 days and the phantom heights were precise. In previous work [20], we showed that the GE unit exhibited serial drift with respect to the BL dataset and drift should be accounted for to maintain prospective calibration accuracy. Because the data in this report was collected over a relatively short time interval, serial drift influences are likely minimal. Similarly, the calibration accuracy was evaluated without height uncertainty. Therefore, the accuracies obtained in this report may be considered ideal.

Our original objective was to develop a continuous calibrated breast density measurement applicable across imaging platforms. Additionally, calibration may be useful for other than risk applications, such as estimating the BI-RADS breast composition descriptors [23]. The BI-RADS breast composition descriptors were developed for standardized reporting purposes and synchronized with situations where mammographic sensitivity may be lower due to composition. Calibrated tissue composition measurements may be useful for both breast cancer risk applications as well as providing quantitative sensitivity measure.

## Conclusion

This initial evaluation in combination with our previous calibration findings indicate that the same calibration approach may apply to both indirect and direct x-ray conversion technologies. Because the BL dataset requires a considerable amount of phantom imaging, it is not cost-effective to acquire serial replications of the BL dataset on a regular basis for calibration purposes. Therefore, it is imperative to evaluate the forward serial applicability or stability of the BL datasets [20]. In addition, alternative methods of updating the BL dataset with a minimal amount of serial phantom imaging will be explored in future work. Previously, we adapted the Cumulative Sum approach to monitor the forward stability of the BL dataset [20]. However, the serial updating remains an open-ended problem. For this report, the compressed breast thickness was not a source of uncertainty. The calibration accuracies in the work were obtained under relatively ideal conditions by design. The compression paddle on the Hologic systems in this report is spring tensioned and therefore somewhat different from the technology we evaluated previously. During actual breast imaging, the compression paddle tilts and warps, and the system compressed breast thickness readout is often nominal [21], which are common traits across the FFDM designs. Additional work is required to assess the influence of uncertainty in paddle height (relative to breast support surface) using deformable phantoms and generate a compressed breast thickness correction before applying calibration to actual mammograms. Although the calibration accuracies were within our preset tolerances for the most part, the viability of our technique with this particular FFDM technology will require evaluation with patient images to show that a calibrated measure of breast density is associated with breast cancer.

## Abbreviations

a: Index reserved for adipose breast tissue equivalent material; Ag: Silver; b: Intercept of the open detector exposure relationships; B: Calibration application additive parameter; BL: Baseline; BTE: Breast tissue equivalent; El(rs): Mean exposure at given sub-region r_s_ and baseline phantom height in cm; FFDM: full field digital mammography; FOV: field of view; g: Index reserved for fibroglandular breast tissue equivalent material; GE: General Electric Senographe 2000D FFDM unit; Glandular: Fibroglandular; H1: Hologic Selenia unit 1; H2: Hologic Selenia unit 2; H3: Hologic Selenia unit 3; k: Index designating a sampled height; l: Subscript index reserved for breast tissue equivalent material; LCC: Left cranial caudal; LIl: Logarithmic intercept; LRE: Natural logarithm of the relative exposure; LREl (rs): Natural logarithm of the relative exposure at r_s_ as a function of increasing baseline phantom height in cm; m: Slope of the open detector exposure relationships; M: Calibration application multiplier factor; mAs: Milliampere × second; Mo: Molybdenum; PG: Percent glandular; PGcal: A calibrated quantity; pva: Adipose pixel value in the LRE domain; pvg: Glandular pixel value in the LRE domain; pvraw: Raw image pixel value; rs: Sub-regions; R2: Coefficient of determination; REl(rs): Relative mean exposure at a given sub-region r_s_ and baseline phantom height in cm; Rh: Rhodium; ROI: Region of interest; SE: Standard error; SD: Standard deviation; tk: Baseline phantom sample height in cm; μl: Effective x-ray attenuation coefficient in cm^-1^; W: Tungsten; x: Arbitrary mAs quantity; xr: The reference, 160 mAs.

## Competing interests

The authors declare that they have no competing interests.

## Authors’ contributions

The database was constructed by EF under the supervision of JH. JH, EF and BL developed the manuscript content. EF, JH and BL performed the data analysis. All authors contributed to manuscript composition. All authors read and approved the final manuscript.
